# Tobacco control needs a choice-based approach to curb cigarette smoking

**DOI:** 10.1016/j.amsu.2022.104186

**Published:** 2022-07-13

**Authors:** Yusuff Adebayo Adebisi, Nafisat Dasola Jimoh, Isaac Olushola Ogunkola, Amusile Olayemi, Awolola Timileyin Omolayo, Damilola Oyedokun

**Affiliations:** Faculty of Pharmacy, University of Ibadan, Ibadan, Nigeria; Federal University of Technology, Minna, Nigeria; Department of Public Health, University of Calabar, Calabar, Nigeria; Federal Medical Centre (FMC), Asaba, Delta State, Nigeria; Faculty of Pharmacy, University of Ibadan, Ibadan, Nigeria

**Keywords:** Tobacco control, Tobacco harm reduction, Rights-based approach, Health policy, Smoking cessation

Dear Editor,

Tobacco control efforts are almost ineffective in most parts of the world [1]. This is concerning because cigarette smoking still claims millions of lives each year, and vulnerable low-and-middle-income countries (LMICs) bear a disproportionate share of the burden (in terms of health inequalities, mortality, and morbidity). A recent editorial expressed concern about the low adoption of a comprehensive package of WHO Framework Convention on Tobacco Control (FCTC) and MPOWER measures [1]. However, statistical evidence has shown that global cigarette consumption trends have not changed substantially after the FCTC's adoption in 2003 [2]. The same evidence reveals that tobacco consumption has actually increased in LMICs [2]. This reifies the need for intensive pragmatic efforts to curb cigarette smoking. Herein, we add our voice to the global discussion on how a choice-based, harm reduction approach can accelerate international efforts to curb cigarette smoking.

The current tobacco control measures have extensively promoted the abstinence-only (quit or die) approach to curb smoking [3]. This has contributed to smokers' inability to make informed choices about other nicotine products that have been shown to be safer [4]. Harm reduction is a public health strategy that is compassionate, people-centered, choice-focused, and rights-based. Despite mounting evidence of its benefits, tobacco control efforts have failed to respect the principle of harm reduction. Interestingly, this denial of harm reduction in practice contradicts the FCTC glossary definition of tobacco control. According to the FCTC (Article 1d), tobacco control means “a range of supply, demand, and *harm reduction strategies* that aim to improve the health of a population by eliminating or reducing their consumption of tobacco products and exposure to tobacco smoke” [5]. Tobacco control efforts have taken a prohibitionist and abstinence-only approach, making it difficult for smokers to obtain reliable and evidence-based information on other nicotine delivery alternatives in order for them to make informed choices.

HIV is an example of a global pandemic where the use of harm reduction tools (condoms) has led to a major achievement in its response. Imagine if there was no choice other than abstinence from sex. The HIV prevalence and incidence would have been higher than the current global rates. With the increase in access to condoms and comprehensive sexual education, evidence has shown no increase in sexual activity among adolescents [6]. Even though there are concerns regarding youth vaping, the prevalence is low and the use is mostly experimental, and should not deter the goal of smoking cessation [3], which reduced-risk nicotine products provide an opportunity to achieve. In tobacco control efforts, access to risk-reduced nicotine delivery options, as well as evidence-based public health education on their risks and benefits, is critical.

Tobacco harm reduction products are worth exploring because they can serve as a method of smoking cessation, as an alternative for new generations of tobacco-prone youth who would otherwise take up smoking, and as an alternative to cigarettes for smokers who are unable or unwilling to quit smoking altogether. A rational, people-centered public health strategy must recognize that risky behavior cannot be completely eliminated. There is always a need for choices whose benefits outweigh any potential risks/harms. No pharmaceutical drug is 100% risk-free. It makes sense to use them in providing medical care because the benefits outweigh the risks or any potential harms. This holds true for the effectiveness of harm reduction products in curbing cigarette smoking.

The principle of harm reduction in quitting cigarette smoking practically respects the role of alternative nicotine delivery methods such as vaping [3]. Yet, the FCTC fails to embrace the role of 'safer' alternative nicotine products in its approach. It is even more concerning that, through tobacco control efforts, countries continue to spread disinformation and misinformation regarding alternative nicotine products. This has infringed on smokers' right to information about these products. The United Kingdom (UK), as a leader in embracing the concept of tobacco harm reduction, has revealed how the use of alternative nicotine products can downtrend smoking rates [7]. The effort to end tobacco smoking in the UK embraces the essential role of alternative nicotine delivery systems, such as e-cigarettes. A study in the UK that examined the use of e-cigarettes across the population estimated that e-cigarettes helped an additional 18,000 people in England in 2015 to quit for the long term [8]. From 2020 to 2021, the use of e-cigarettes in conjunction with local stop-smoking services resulted in significant successful quitting rates in the UK [9]. In Sweden and Norway, snus availability has contributed to the unusually low prevalence of tobacco smoking by providing options to switch to a notably less harmful form of nicotine dependence [10].

The benefit of tobacco harm reduction is scientific. According to Public Health England, e-cigarettes are 95% safer than cigarette smoking [11]. Yet, the current tobacco control effort continues to deny smokers the right to make a choice and to have access to complete, unbiased information regarding tobacco harm reduction. It is high time countries around the world embraced tobacco harm reduction. It is not only rights-based; it provides the opportunity to eradicate cigarette smoking quickly and addresses all inherent health inequalities. Tobacco harm reduction products are not without risk, but it is beneficial for smokers, particularly those who are unable or unwilling to quit smoking, to switch to a lower-risk nicotine product. Policies aimed at limiting access to tobacco harm reduction products may have (inadvertently) increased cigarette smoking.

We urge countries to embrace a choice-based approach to ending cigarette smoking, one that acknowledges switching to alternative nicotine products as a strategy to curb smoking. This is urgently needed because millions of people's lives are at stake.

## Disclaimer

The views presented in this piece are of the authors and do not necessarily represent the official position of their institutions.

## Ethical approval

Not Required.

## Sources of funding

None.

## Author contribution

Yusuff Adebayo Adebisi conceptualized the paper. Nafisat Dasola Jimoh and Yusuff Adebayo Adebisi wrote the first draft. Isaac Olushola Ogunkola, Amusile Olayemi, Awolola Timileyin Omolayo, and Damilola Oyedokun contributed to the first draft and revised it. All the authors approved the paper for publication.

## Conflicts of interest

Yusuff Adebayo Adebisi is a Kevin Molloy Fellow, under the tobacco harm reduction scholarships programme by Knowledge.Action.Change UK. Nafisat Dasola Jimoh and Isaac Olushola Ogunkola were also a recipient of tobacco harm reduction scholarships from the same organization. The fellowship and the scholarship are focused on building capacity in the field of tobacco harm reduction. Only the authors are involved in conceptualizing and writing the content of this article, it is independent of Knowledge. Action.Change UK.

## Registration of research studies

1. Name of the registry: Not Applicable.

2. Unique Identifying number or registration ID: Not Applicable.

3. Hyperlink to your specific registration (must be publicly accessible and will be checked): Not Applicable.

## Guarantor

Yusuff Adebayo Adebisi.

## Consent

Not required.
